# Traxoprodil, a selective antagonist of the NR2B subunit of the NMDA receptor, potentiates the antidepressant-like effects of certain antidepressant drugs in the forced swim test in mice

**DOI:** 10.1007/s11011-016-9810-5

**Published:** 2016-02-29

**Authors:** Ewa Poleszak, Weronika Stasiuk, Aleksandra Szopa, Elżbieta Wyska, Anna Serefko, Anna Oniszczuk, Sylwia Wośko, Katarzyna Świąder, Piotr Wlaź

**Affiliations:** Department of Applied Pharmacy, Medical University of Lublin, Chodźki 1, PL 20-093 Lublin, Poland; Department of Human Physiology, Medical University of Lublin, Lublin, Poland; Department of Pharmacokinetics and Physical Pharmacy, Collegium Medicum, Jagiellonian University, Kraków, Poland; Department of Inorganic Chemistry, Medical University of Lublin, Lublin, Poland; Department of Animal Physiology, Institute of Biology and Biochemistry, Faculty of Biology and Biotechnology, Maria Curie-Skłodowska University, Lublin, Poland

**Keywords:** Traxoprodil, Antidepressants, Forced swim test, Pharmacokinetic study, Mice

## Abstract

One of the newest substances, whose antidepressant activity was shown is traxoprodil, which is a selective antagonist of the NR2B subunit of the NMDA receptor. The main goal of the present study was to evaluate the effect of traxoprodil on animals’ behavior using the forced swim test (FST), as well as the effect of traxoprodil (10 mg/kg) on the activity of antidepressants, such as imipramine (15 mg/kg), fluoxetine (5 mg/kg), escitalopram (2 mg/kg) and reboxetine (2.5 mg/kg). Serotonergic lesion and experiment using the selective agonists of serotonin receptors 5-HT_1A_ and 5-HT_2_ was conducted to evaluate the role of the serotonergic system in the antidepressant action of traxoprodil. Brain concentrations of tested agents were determined using HPLC. The results showed that traxoprodil at a dose of 20 and 40 mg/kg exhibited antidepressant activity in the FST and it was not related to changes in animals’ locomotor activity. Co-administration of traxoprodil with imipramine, fluoxetine or escitalopram, each in subtherapeutic doses, significantly affected the animals’ behavior in the FST and, what is important, these changes were not due to the severity of locomotor activity. The observed effect of traxoprodil is only partially associated with serotonergic system and is independent of the effect on the 5-HT_1A_ and 5-HT_2_ serotonin receptors. The results of an attempt to assess the nature of the interaction between traxoprodil and the tested drugs show that in the case of joint administration of traxoprodil and fluoxetine, imipramine or escitalopram, there were interactions in the pharmacokinetic phase.

## Introduction

Glutamate (Glu) is one of the most important and present in the highest concentration excitatory amino acid neurotransmitter in the central nervous system (CNS) (McGeer et al. 1987). Glutamatergic system has the greatest diversity of both construction and function of receptors compared with other CNS neurotransmitter systems. Glutamate ionotropic receptors have been identified as the first ones and, in the 80s, the first scientific evidence on metabotropic receptors was provided (Nicoletti et al. 1986). One of the ionotropic glutamate receptors is NMDA receptor, which is stimulated by N-methyl-d-aspartic acid (NMDA) (Glasgow et al. 2015; Machado-Vieira et al. 2010; Traynelis et al. 2010).

In the 1990s the first studies showing the antidepressant-like effect of compounds that are antagonists of NMDA receptors were carried out (Trullas and Skolnick 1990). Nowadays, there are a number of pre-clinical and clinical reports which have shown the antidepressant potential of the NMDA receptor antagonists, as well as their influence on the effectiveness of the antidepressant drugs (Cichy et al. 2009; Dybała et al. 2008; Heresco-Levy et al. 2006; Muhonen et al. 2008; Poleszak et al. 2008; Preskorn et al. 2008; Skolnick et al. 2009; Sowa-Kućma et al. 2011; Szewczyk et al. 2009, 2010; Zarate et al. 2006). Therefore, inhibition of the NMDA receptor complex may provide new possibilities in the treatment of mental disorders (e.g., DiazGranados et al. 2010; Gosek et al. 2012; Maeng and Zarate 2007; Poleszak et al. 2014; Price et al. 2009).

The role of NR2B subunit of the NMDA receptor has been demonstrated in the action of different antidepressant agents (Layer et al. 1995; Li et al. 2011; Maeng et al. 2008; Poleszak et al. 2013, 2014; Preskorn et al. 2008). A number of clinical studies have confirmed the remarkable antidepressant effects produced by the NMDA antagonist – traxoprodil (CP-101,606) (Preskorn et al. 2008; Skolnick et al. 2009; Zarate et al. 2006). Traxoprodil is a selective antagonist of the NR2B subunit of the NMDA receptor (Chenard et al. 1995). It antagonizes the activity of the NR1/NR2B channel by shortening the time and frequency of its opening. As a result, it prevents a damaging influx of calcium ions into the neurons caused by the release of large quantities of glutamate from the damaged tissue (Kundrotiene et al. 2004; Mony et al. 2009). Traxoprodil binding site is mainly located in forebrain, hippocampus and the outer layers of cortex (Menniti et al. 1997). This agent appeared to be safe and generally well-tolerated, capable of producing an antidepressant response in patients with treatment-refractory major depressive disorders (Preskorn et al. 2008).

The main goal of this study was to assess the effect of traxoprodil on animals’ behavior using the forced swim test (FST) in mice. Moreover, we also decided to evaluate the influence of traxoprodil at the inactive dose on the activity of the commonly used antidepressants, i.e., imipramine – a tricyclic antidepressant (TCA), fluoxetine, escitalopram – a selective serotonin reuptake inhibitor (SSRI), and reboxetine – a selective noradrenaline reuptake inhibitor (SNRI). In order to evaluate the role of the serotonergic system in the antidepressant potential of traxoprodil, we subjected the mice to serotonergic lesion with p-chlorophenylalanine (p-CPA). In order to elucidate the role of serotonin receptors 5-HT_1A_ and 5-HT_2_ in the operation of traxoprodil, we conducted experiment using the selective agonists of these receptors – WAY 100,635 and ritanserin, respectively.

## Materials and methods

### Animals

The experiments were carried out on naïve adult male Albino Swiss mice (25–30 g) purchased from the licensed breeder (Kołacz, Warsaw, Poland). The animals were housed in the environmentally controlled rooms with a 12 h light/dark cycle, in groups of 10 in standard cages under strictly controlled laboratory conditions – temperature maintained at 22–23 °C, relative humidity about 45–55 %. Throughout the study, the animals were given ad libitum access to water and food. The experiments began after at least 1-week acclimation period in the laboratory conditions and were conducted between 8 a.m. and 3 p.m. to minimize circadian influence. Each experimental group consisted of 8–10 animals. All procedures were conducted in accordance with the European Communities Council Directive of 22 September 2010 (2010/63/EU) and Polish legislation acts concerning animal experimentations. The experimental procedures and protocols were approved by the First Local Ethics Committee at the Medical University of Lublin (license no 33/2013). Each mouse was used only once.

### Drug administration

Traxoprodil (5, 10, 20, and 40 mg/kg, Sigma-Aldrich) was suspended in a 1 % aqueous solution of Tween 80 (POCH), whereas imipramine hydrochloride (15 and 30 mg/kg, Sigma-Aldrich), fluoxetine hydrochloride (5 mg/kg, Sigma-Aldrich), escitalopram oxalate (2 mg/kg, Sigma-Aldrich), reboxetine mesylate (2.5 mg/kg, Abcam Biochemicals), WAY 100,635 (0.1 mg/kg, Sigma-Aldrich), and ritanserin (4 mg/kg, Sigma-Aldrich) were dissolved in physiological saline (0.9 % NaCl). The solutions/suspension were prepared immediately prior to the experiments and were administered intraperitoneally (i.p.) 60 min before testing. The doses and pretreatment schedules were selected on the basis of the literature data and the results of our previous experiments (Poleszak et al. 2005, 2007a, 2011, 2013; Szewczyk et al. 2002, 2009). Animals from the control groups received i.p. injections of the vehicle (saline). The volume of all administered solutions/suspension was 10 ml/kg.

### Serotonergic lesion

p-CPA was dissolved in saline and administered i.p. at a dose of 200 mg/kg for 3 consecutive days. Mice from the control group received i.p. injections of saline. On the fourth day, the animals were given traxoprodil at an active dose (20 mg/kg) or saline, and 60 min later, the FST and locomotor activity tests were performed.

### Forced swim test (FST)

The procedure was carried out on mice, according to the method of Porsolt et al. (1977). Each mouse was placed individually into a glass cylinder (height 25 cm, diameter 10 cm) containing 12–15 cm of water at 23–25 °C. The animal was left in the cylinder for 6 min. The total duration of immobility was recorded during the last 4 min of the 6-min testing period. The mouse was judged to be immobile when it ceased struggling and remained floating motionless in the water, making only the movements necessary to keep its head above the water level.

The results obtained in the FST were shown as an arithmetic mean of immobility time of animals (given in seconds) ± standard error of the mean (SEM) for each experimental group.

### Spontaneous locomotor activity

In order to avoid the risk of obtaining the false positive/negative effects in the FST caused by a possible influence of the tested drugs on the locomotor activity, spontaneous locomotor activity was measured using an animal activity meter Opto-Varimex-4 Auto-Track (Columbus Instruments, USA). The device consists of four transparent cages with a lid (43 × 43 × 32 cm), a set of four infrared emitters (each emitter has 16 laser beams), and four detectors monitoring animal movements. Each mouse was placed individually into the cage for 10 min. Spontaneous locomotor activity was evaluated between the 2nd and the 6th min, which corresponds with the time interval analyzed in the FST.

The results obtained in this test were presented as an arithmetic average distance (given in cm) traveled by a mouse ± SEM for each experimental group.

### Determination of antidepressants and traxoprodil in brains

Sixty minutes following administration of studied antidepressant drugs with or without traxoprodil, mice were decapitated to collect brains for pharmacokinetic studies. Immediately after the decapitation, the brains were dissected from the skull, washed with 0.9 % NaCl and frozen at −25 °C.

Brain concentrations of the studied antidepressants and traxoprodil were assayed by high performance liquid chromatography (HPLC) methods. The brains were homogenized in distilled water (1 : 4, w/v) with a tissue homogenizer TH220 (Omni International, Inc., Warrenton, VA, USA). For all studied antidepressant drugs, the extraction from brain homogenates were performed using the mixture of ethyl acetate : hexane (30 : 70, *v*/*v*). Amitriptyline (2 μg/ml) was used as an internal standard (IS) for imipramine and paroxetine (200 ng/ml) for fluoxetine and escitalopram. In order to isolate imipramine and its metabolite desipramine, to brain homogenate (0.5 ml) containing these drugs the IS was added and the samples were alkalized with 250 μl of 4 M NaOH. Then the samples were extracted with 5 ml of the extraction reagent by shaking for 20 min (IKA Vibrax VXR, Germany). After centrifugation at 3000 rpm for 20 min (Universal 32, Hettich, Germany), the organic layer was transferred to a new tube containing a 200 μl solution of 0.1 M H_2_SO_4_ and methanol (90 : 10, *v*/*v*), shaken for 0.5 h and then centrifuged for 15 min (3000 rpm). The organic layer was discarded and a 50 μl aliquot of the acidic solution was injected into the HPLC system. In the case of escitalopram, the procedure was similar with the exception that the extraction with an organic reagent was repeated two times, 1 ml of brain homogenate was used, and the volume of the acidic phase was 100 μl. In turn, to 1 ml of brain homogenates containing fluoxetine the IS was added and the samples were alkalized with 500 μl of 4 M NaOH. After the addition of 1 ml of the concentrated NaCl solution (10 g/50 ml), the samples were vortexed for 15 s and 5 ml of the extraction reagent was added. Then the samples were shaken for 20 min and centrifuged for 15 min at 3000 rpm. After the centrifugation, the organic layer was transferred into a conical glass tube and evaporated to dryness at 37 °C under a gentle stream of nitrogen in a water bath. The residue was dissolved with 100 μl of methanol and 50 μl of this solution was injected into the HPLC system.

The HPLC system (Thermo Separation Products, San Jose, CA, USA) consisted of a P100 isocratic pump, a UV100 variable-wavelength UV/VIS detector, a Rheodyne 7125 injector (Rheodyne, Cotati, CA, USA) with a 50 μl sample loop, and a Chromjet SP4400 computing integrator.

All analyses were performed on a 250 × 4.0 mm LiChrospher®100 RP-18 column with a particle size of 5 μm (Merck, Darmstadt, Germany) protected with a guard column (4 × 4 mm) with the same packing material. The mobile phase consisting of 50 mM potassium dihydrogen phosphate buffer (pH = 4.5) and acetonitrile was mixed at a ratio of 60 : 40 (*v*/*v*) for imipramine and fluoxetine, and 65 : 35 (*v*/*v*) for escitalopram and run at 1 ml/min. Chromatographic analysis was carried out at 21 °C and the analytical wavelength of 227 nm for fluoxetine, 240 nm for escitalopram, and 214 nm for imipramine.

In order to determine traxoprodil concentrations in mice brain, to 1 ml of brain homogenate containing this compound 2 ml of methanol was added and the samples were briefly vortexed and then shaken vigorously for 10 min (IKA Vibrax VXR, Germany) to precipitate proteins. After centrifugation for 20 min at 3000 rpm the supernatant (2 ml) was transferred into a conical glass tube and evaporated to dryness at 45 °C under a gentle stream of nitrogen in a water bath. The residue was dissolved with 100 μl of methanol and 40 μl of this solution were injected into the HPLC system.

The HPLC system (Merck-Hitachi LaChrom Elite) consisted of an L-2130 pump, an L-2200 autosampler, an L-2350 column oven, and an L-2485 fluorescence detector. EZChrome Elite v. 3.2 (Merck Hitachi) software was used for data acquisition. The analysis was performed on a 250 × 4.0 mm LiChrospher®100 RP-18 column (Merck, Darmstadt, Germany) maintained at 30 °C, protected with a guard-column (4 × 4 mm) of the same material. The mobile phase consisted of 50 mM potassium dihydrogen phosphate buffer, pH 4.5 : acetonitrile : methanol (70:20:10, *v*/*v*/*v*). The flow rate was 1.0 ml/min and the fluorescence detector was set at an excitation wavelength of 200 nm and an emission wavelength of 300 nm.

The calibration curves constructed by plotting the ratio of the peak heights of the studied drug to IS (or peak area for traxoprodil) versus concentration of the drug were linear in the tested concentration ranges. No interfering peaks were observed in the chromatograms. The assays were reproducible with low intra- and inter-day variation (coefficient of variation less than 10 %). The extraction efficiencies of the analyzed compounds and internal standards ranged from 66 to 97 %. Concentrations of antidepressants and traxoprodil were expressed in ng/g of wet brain tissue.

### Statistical analysis

The statistical analysis of the results obtained in the FST and the locomotor activity assessment following traxoprodil administration was carried out using one-way ANOVA with Dunnett’s post hoc test and after joint treatments using two-way ANOVA with Bonferroni’s post hoc test. The concentrations of the tested antidepressant drugs in murine brains in the presence and absence of traxoprodil were compared using Student’s *t*-test. P values less than or equal to 0.05 were considered statistically significant.

## Results

### Forced swim test (FST)

#### Traxoprodil dose-effect relationship in FST

**Fig. 1:**
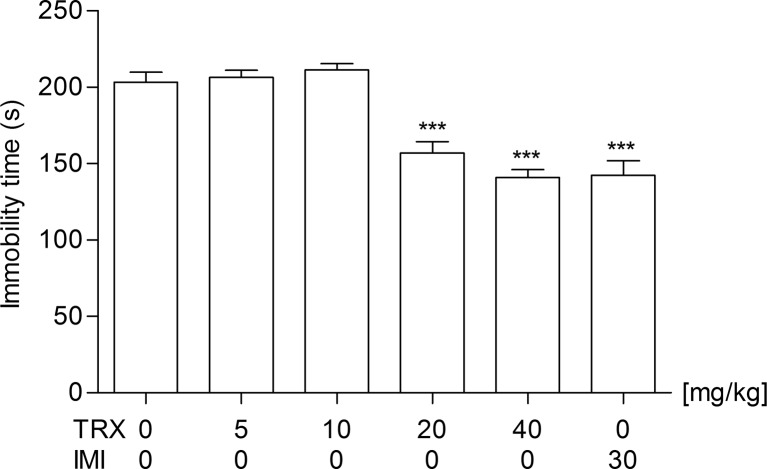
The antidepressant activity of traxoprodil in the FST in mice. Traxoprodil, imipramine and saline were administered i.p. 60 min before the test. The data are presented as the means + SEM. Each experimental group consisted of 8 animals. ***p < 0.001 (one-way ANOVA followed by Dunnett’s post hoc test)

In order to determine an antidepressant activity of traxoprodil, it was used at doses of 5, 10, 20, and 40 mg/kg (Fig. 1). Statistical analysis of the results obtained in the FST showed that traxoprodil used at doses of 5 and 10 mg/kg had no statistically significant effect (*p* > 0.05) on the reduction of the immobility time in mice. However, traxoprodil administered at a dose of 20 and 40 mg/kg significantly reduced the total time of immobility in comparison with the control group [one-way ANOVA: F(5,42) = 26.41; *p* < 0.0001].

#### Effect of combined administration of traxoprodil and imipramine in FST

**Fig. 2:**
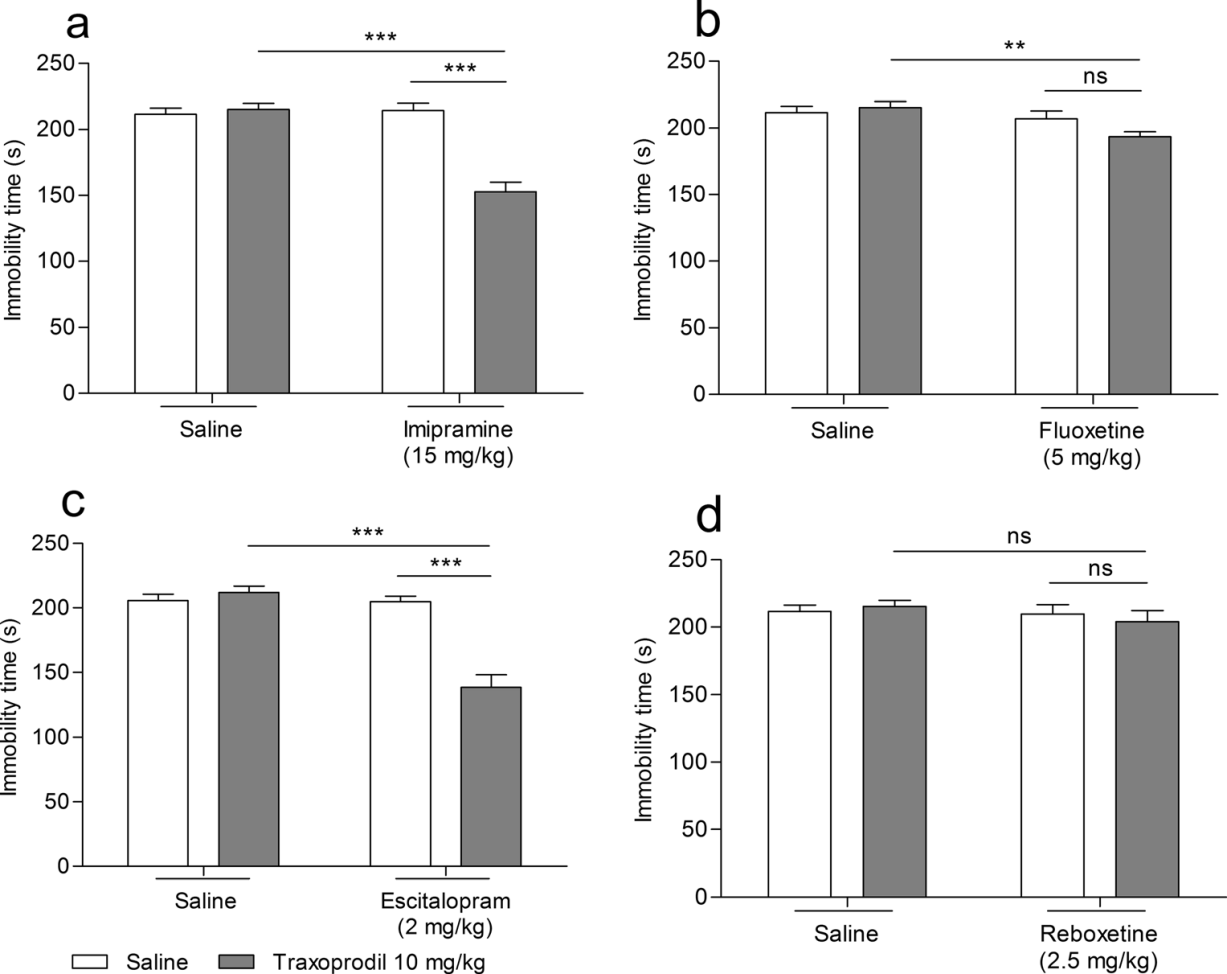
Effect of combined administration of traxoprodil and antidepressants in the FST in mice. Antidepressants, traxoprodil and saline were administered i.p. 60 min before the test. The values represent mean + SEM (*n* = 10 per group). **p < 0.01; ***p < 0.001 (two-way ANOVA followed by Bonferroni’s post hoc test)

The effect of the combined administration of traxoprodil and imipramine on total duration of the immobility time in mice is shown in Fig. 2a. Traxoprodil (10 mg/kg) injected in combination with imipramine (15 mg/kg) significantly reduced the immobility time in the FST in mice (Fig. 2a). Imipramine (15 mg/kg) and traxoprodil (10 mg/kg) given alone had no effect on the immobility time (Fig. 2a).

Two-way ANOVA demonstrated a significant effect of traxoprodil [F(1,28) = 26.08; *p* < 0.0001], a significant effect of imipramine [F(1,28) = 27.44; *p* < 0.0001], and a significant interaction between imipramine and traxoprodil [F(1,28) = 32.99; *p* < 0.0001].

#### Effect of combined administration of traxoprodil and fluoxetine in FST

The effect of the combined administration of traxoprodil and fluoxetine on total duration of the immobility time in mice is shown in Fig. 2b. Traxoprodil (10 mg/kg) injected in combination with fluoxetine (5 mg/kg) significantly reduced the immobility time in the FST in mice (Fig. 2b). Fluoxetine (5 mg/kg) and traxoprodil (10 mg/kg) given alone had no effect on the immobility time (Fig. 2b).

Two-way ANOVA demonstrated no effect of traxoprodil [F(1,28) = 1.05; *p* = 0.3151], a significant effect of fluoxetine [F(1,28) = 7.46; *p* = 0.0108], and no interaction between fluoxetine and traxoprodil [F(1,28) = 3.15; *p* = 0.0870].

#### Effect of combined administration of traxoprodil and escitalopram in FST

The effect of the combined administration of traxoprodil and escitalopram on total duration of the immobility time in mice is shown in Fig. 2c. Traxoprodil (10 mg/kg) injected in combination with escitalopram (2 mg/kg) significantly reduced the immobility time in the FST in mice (Fig. 2c). Escitalopram (2 mg/kg) and traxoprodil (10 mg/kg) given alone had no effect on the immobility time (Fig. 2c).

Two-way ANOVA demonstrated a significant effect of traxoprodil [F(1,28) = 22.79; *p* < 0.0001], a significant effect of escitalopram [F(1,28) = 34.90; *p* < 0.0001], and a significant interaction between escitalopram and traxoprodil [F(1,28) = 33.27; *p* < 0.0001].

#### Effect of combined administration of traxoprodil and reboxetine in FST

The effect of the combined administration of traxoprodil and reboxetine on total duration of the immobility time in mice is shown in Fig. 2d. Traxoprodil (10 mg/kg) injected in combination with reboxetine (2.5 mg/kg) did not reduce the immobility time in the FST in mice (Fig. 2d). Reboxetine (2.5 mg/kg) and traxoprodil (10 mg/kg) given alone had no effect on the immobility time (Fig. 2d).

Two-way ANOVA demonstrated no effect of traxoprodil [F(1,28) = 0.02; *p* = 0.8827], no effect of reboxetine [F(1,28) = 1.09; *p* = 0.3061], and no interaction between reboxetine and traxoprodil [F(1,28) = 0.53; *p* = 0.4746].

#### Influence of serotonergic lesion on antidepressant-like activity of traxoprodil in FST

**Fig. 3:**
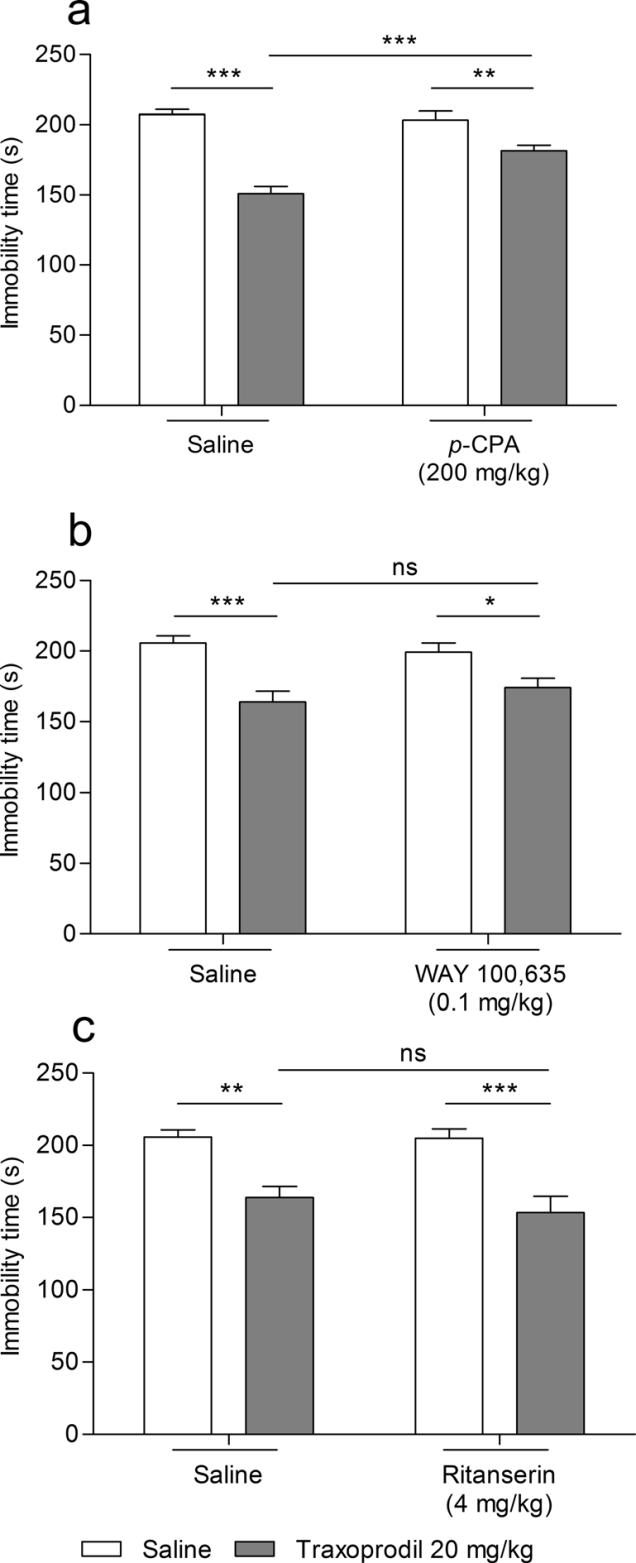
Effect of combined administration of traxoprodil and p-CPA and selective agonists of serotonin receptors 5-HT_1A_ and 5-HT_2_ in the FST in mice. Traxoprodil, WAY 100635, ritanserin and saline were administered i.p. 60 min before the test. p-CPA was administered i.p. once per day over three consecutive days. The values represent mean + SEM (*n* = 10 per group). *p < 0.05; **p < 0.01; ***p < 0.001 (two-way ANOVA followed by Bonferroni’s post hoc test)

The effect of the combined administration of traxoprodil and p-CPA on total duration of the immobility time in mice is shown in Fig. 3a. Traxoprodil (20 mg/kg) significantly reduced the immobility time in the FST in mice (Fig. 3a). p-CPA (200 mg/kg administered per 3 days) had no effect on the immobility time (Fig. 3a). Traxoprodil (20 mg/kg) injected in combination with p-CPA (200 mg/kg administered per 3 days) partially reversed the antidepressant-like effect of traxoprodil in the FST in mice (Fig. 3a).

Two-way ANOVA demonstrated a significant effect of traxoprodil [F(1,28) = 66.37; *p* < 0.0001], a significant effect of p-CPA [F(1,28) = 7.80; *p* = 0.0093], and a significant interaction between p-CPA and traxoprodil [F(1,28) = 12.95; *p* = 0.0012].

#### Influence of WAY 100,635 on antidepressant-like activity of traxoprodil in FST

The effect of the combined administration of traxoprodil and WAY 100,635 on total duration of the immobility time in mice is shown in Fig. 3b. Traxoprodil (20 mg/kg) significantly reduced the immobility time in the FST in mice (Fig. 3b). WAY 100,635 (0.1 mg/kg) had no effect on the immobility time (Fig. 3b). Traxoprodil (20 mg/kg) injected in combination with WAY 100,635 (0.1 mg/kg) did not reverse the antidepressant-like effect of traxoprodil (20 mg/kg) in the FST in mice versus group receiving traxoprodil, but significantly reduced the immobility time versus group receiving WAY 100,635 (0.1 mg/kg) (Fig. 3b).

Two-way ANOVA demonstrated a significant effect of traxoprodil [F(1,27) = 26.49; *p* < 0.0001], no effect of WAY 100,635 [F(1,27) = 0.08; *p* = 0.7745], and no interaction between WAY 100,635 and traxoprodil [F(1,27) = 1.66; *p* = 0.2087].

#### Influence of ritanserin on antidepressant-like activity of traxoprodil in FST

The effect of the combined administration of traxoprodil and ritanserin on total duration of the immobility time in mice is shown in Fig. 3c. Traxoprodil (20 mg/kg) significantly reduced the immobility time in the FST in mice (Fig. 3c). Ritanserin (4 mg/kg) had no effect on the immobility time (Fig. 3c). Traxoprodil (20 mg/kg) injected in combination with ritanserin (4 mg/kg) did not reverse the antidepressant-like effect of traxoprodil (20 mg/kg) in the FST in mice versus group receiving traxoprodil, but significantly reduced the immobility time versus group receiving ritanserin (4 mg/kg) (Fig. 3c).

Two-way ANOVA demonstrated a significant effect of traxoprodil [F(1,27) = 33.67; *p* < 0.0001], no effect of ritanserin [F(1,27) = 0.50; *p* = 0.4864], and no interaction between ritanserin and traxoprodil [F(1,27) = 0.36; *p* = 0.5555].

### Spontaneous locomotor activity

#### Effect of traxoprodil on locomotor activity in mice

The effect of traxoprodil (5, 10, 20, and 40 mg/kg) on spontaneous locomotor activity in mice is shown in Table 1. Statistical analysis of the results showed that traxoprodil used in all tested doses had no statistically significant effect on locomotor activity in mice versus control group [one-way ANOVA: F(5,42) = 1.661; *p* = 0.1653].

**Table 1:** Effect of traxoprodil on locomotor activity in mice

Treatment (mg/kg)	Distance traveled (cm)
saline (control group)	1014 ± 151.8
traxoprodil 5	915.0 ± 92.10
traxoprodil 10	738.5 ± 49.93
traxoprodil 20	912.0 ± 75.87
traxoprodil 40	789.1 ± 119.8
imipramine 30	653.9 ± 99.38

Traxoprodil, imipramine and saline were administered i.p. 60 min before the test. Distance traveled was recorded between the 2nd and the 6th min of the test. The data are presented as the means ± SEM. Each experimental group consisted of 8 animals. The results were considered statistically significant if *p* < 0.05 (one-way ANOVA followed by Dunnett’s post hoc test)

#### Effect of combined administration of traxoprodil and antidepressants on locomotor activity in mice

The effect of the combined administration of traxoprodil and tested antidepressant drugs on spontaneous locomotor activity in mice is shown in Table 2.

**Table 2:** Effect of treatments on spontaneous locomotor activity in mice

	Treatment (mg/kg)	Distance traveled (cm)
(A)	saline + saline	705.0 ± 39.07
	traxoprodil 10 + saline	629.1 ± 79.29
	imipramine 15 + saline	492.3 ± 57.11
	traxoprodil 10 + imipramine 15	638.3 ± 57.77
(B)	saline + saline	705.0 ± 39.07
	traxoprodil 10 + saline	629.1 ± 79.29
	fluoxetine 5 + saline	667.8 ± 111.1
	traxoprodil 10 + fluoxetine 5	728.4 ± 96.91
(C)	saline + saline	815.6 ± 63.98
	traxoprodil 10 + saline	986.8 ± 278.5
	escitalopram 2 + saline	957.1 ± 174.1
	traxoprodil 10 + escitalopram 2	1096 ± 107.6
(D)	saline + saline	705.0 ± 39.07
	traxoprodil 10 + saline	629.1 ± 79.29
	reboxetine 2.5 + saline	566.1 ± 54.75
	traxoprodil 10 + reboxetine 2.5	636.0 ± 100.8
(E)	saline + saline	983.8 ± 224.3
	traxoprodil 20 + saline	731.1 ± 145.1
	p-CPA 200 + saline	656.4 ± 107.4
	traxoprodil 20 + p-CPA 200	716.0 ± 70.99
(F)	saline + saline	815.6 ± 63.98
	traxoprodil 20 + saline	687.5 ± 71.45
	WAY 0.1 + saline	639.0 ± 59.16
	traxoprodil 20 + WAY 0.1	615.8 ± 53.48
(G)	saline + saline	815.6 ± 63.98
	traxoprodil 20 + saline	687.5 ± 71.45
	ritanserin 4 + saline	607.5 ± 79.78
	traxoprodil 20 + ritanserin 4	558.9 ± 53.11

Antidepressants, traxoprodil and saline were administered i.p. 60 min before the experiment. p-CPA was administered i.p. once daily over three consecutive days. Distance traveled was recorded between the 2nd and the 6th min of the test. Each experimental group consisted of 8 animals. Data are presented as the means ± SEM. The results were considered statistically significant if *p* < 0.05 (two-way ANOVA followed by Bonferoni’s post hoc test)

Traxoprodil (10 and 20 mg/kg), antidepressants (imipramine, fluoxetine, escitalopram, and reboxetine), or p-CPA, WAY 100,635 and ritanserin administered either alone or combined together had no statistically significant effects on locomotor activity in mice (Table 2).

Two-way ANOVA demonstrated:

(A): no effect of imipramine [F(1,26) = 2.72; *p* = 0.1112], no effect of traxoprodil [F(1,26) = 032; *p* = 0.5752], and no interaction [F(1,26) = 3.23; *p* = 0.0840].
(B): no effect of fluoxetine [F(1,27) = 0.12; *p* = 0.7289], no effect of traxoprodil [F(1,27) = 0.01; *p* = 0.9320], and no interaction [F(1,27) = 0.59; *p* = 0.4473].
(C): no effect of escitalopram [F(1,28) = 0.51; *p* = 0.4817], no effect of traxoprodil [F(1,28) = 0.78; *p* = 0.3855], and no interaction [F(1,28) = 0.01; *p* = 0.9273].
(D): no effect of reboxetine [F(1,27) = 0.79; *p* = 0.3825], no effect of traxoprodil [F(1,27) = 0.01; *p* = 0.9681], and no interaction [F(1,27) = 0.96; *p* = 0.3357].
(E): no effect of p-CPA [F(1,28) = 1.33; *p* = 0.2579], no effect of traxoprodil [F(1,28) = 0.42; *p* = 0.5205], and no interaction [F(1,28) = 1.11; *p* = 0.3014].
(F): no effect of WAY 100,635 [F(1,28) = 3.96; *p* = 0.0563], no effect of traxoprodil [F(1,28) = 1.47; *p* = 0.2351], and no interaction [F(1,28) = 0.71; *p* = 0.4076].
(G): no effect of ritanserin [F(1,28) = 6.17; *p* = 0.0193], no effect of traxoprodil [F(1,28) = 1.70; *p* = 0.2030], and no interaction [F(1,28) = 0.34; *p* = 0.5624].

### Pharmacokinetic studies

The effect of traxoprodil on brain concentrations of the tested antidepressants in mice is shown in Table 3. A significant increase in concentrations of imipramine, its metabolite (desipramine), and escitalopram in brain tissue after joint administration with traxoprodil were noticed (*t*-test: *p* < 0.05, *p* < 0.001, and *p* < 0.05, respectively). In the case of co-administration of traxoprodil and fluoxetine no significant changes in fluoxetine concentration in brain were observed (*t*-test: *p* > 0.05) (Table 3).

**Table 3:** Effect of traxoprodil on the concentration of antidepressants in mouse brain

	Treatment (mg/kg)	Antidepressants concentration in brain (ng/g)
(A)	imipramine 15 + saline	2065 ± 252.6
	(metabolite – desipramine)	(101.0 ± 19.86)
	imipramine 15 + traxoprodil 10	3207 ± 373.8*
	(metabolite – desipramine)	(724.0 ± 109.0***)
(B)	fluoxetine 5 + saline	4835 ± 382.8
	fluoxetine 5 + traxoprodil 10	5122 ± 261.8
(C)	escitalopram 2 + saline	295.5 ± 17.68
	escitalopram 2 + traxoprodil 10	360.9 ± 20.46*

Antidepressants and traxoprodil were administered i.p. 60 min before decapitation. Each experimental group consisted of 10 animals. Results are presented as mean values ± SEM. *p < 0,05; ***p < 0.001 compared with the respective control group (Student’s *t*-test)

The effect of tested drugs on brain concentration of traxoprodil in mice is shown in Table 4. In the case of joint administration of traxoprodil and fluoxetine or escitalopram a significant increase in traxoprodil concentration in brain was noted (*t*-test: *p* < 0.001 and *p* < 0.01, respectively). No statistically significant changes in concentration of traxoprodil were obtained in the group treated with traxoprodil and imipramine *vs* the traxoprodil group (*t*-test: *p* > 0.05).

**Table 4:** Effect of antidepressants on the concentrations of traxoprodil in mouse brain

	Treatment (mg/kg)	Traxoprodil concentration in brain (ng/g)
	traxoprodil 10 + saline	76.40 ± 13.51
(A)	traxoprodil 10 + imipramine 15	119.2 ± 22.52
(B)	traxoprodil 10 + fluoxetine 5	150.6 ± 10.34***
(C)	traxoprodil 10 + escitalopram 2	248.9 ± 49.25**

Antidepressants and traxoprodil were administered *ip* 60 min before decapitation. Each experimental group consisted of 7–8 animals. Results are presented as mean values ± SEM. **p < 0.01; ***p < 0.001 compared with the control group (Student’s *t*-test)

## Discussion

To our knowledge, this is the first study to demonstrate interactions between traxoprodil and antidepressant drugs acting via the monoamine transduction given at non-effective doses in the FST in mice.

The antidepressant activity of the NMDA receptor antagonists has been revealed in many tests and depression models. It was proved that competitive NMDA receptor antagonists (AP7, CGP 37849), zinc ligands (Zn^2+^), polyamine ligands (eliprodil, ifenprodil), phencyclidine ligands (memantine, MK-801) and glycine ligands (ACPC, 7- chlorokynurenic acid) show antidepressant-like activity in a forced swim test (Cichy et al. 2009; Dybała et al. 2006, 2008; Ossowska et al. 1997; Papp and Moryl 1994; Poleszak et al. 2007b, 2008; Redmond et al. 1997; Sowa-Kućma et al. 2008; Szewczyk et al. 2001, 2006, 2008, 2009, 2010). It should be noted that the observed effect was comparable with that of tricyclic antidepressants. In animal studies, it was observed that the abrupt withdrawal of imipramine entails a rapid and significant increase in glutamatergic transmission (Skolnick et al. 1996). It was also found that a variety of ligands that modulate the NMDA complex enhance the effects of antidepressant drugs such as imipramine, citalopram or fluoxetine (Cieślik et al. 2007; Poleszak et al. 2011, 2014; Szewczyk et al. 2002).

One of the newest substances, whose antidepressant activity was shown is CP-101,606 (traxoprodil) (Chazot et al. 2002; Chenard et al. 1995; Menniti et al. 2000). Traxoprodil is an NMDA receptor antagonist with a strong affinity for the NR2B subunit of this receptor (Guscott et al. 2003; Loftis and Janowsky 2003; Menniti et al. 1997, 2000). It is an analogue of ifenprodil, but devoid of activity against α_1_- adrenergic receptors, which eliminates the side effects. By modulating the proton (Dingledine et al. 1999; Guscott et al. 2003; Mott et al. 1998) and allosteric regulation (Mony et al. 2009) traxoprodil inhibits NMDA receptor activity. Traxoprodil, by inhibition of channel activity of subunits NR1/NR2B, reduces the time and the frequency of its opening, thus preventing the excessive influx of calcium ions into neurons, and their damage, and consequently the release of large amounts of glutamic acid (Brimecombe et al. 1998; Chenard et al. 1995).

Recently encouraging results brought the research on applying traxoprodil in the treatment of depression. Its antidepressant effect was similar to that of ketamine, and it brought a bigger relief in depressive manifestations compared with placebo, and a fast improvement in the condition of patients not responding to treatment with SSRIs (Preskorn et al. 2008). In the present study, the antidepressant-like effect of traxoprodil in the FST in mice has been shown. The obtained results demonstrated that a 20 and 40 mg/kg dose of traxoprodil are sufficient to obtain a statistically significant reduction in the immobility time of animals in carried out behavioral tests. The results are consistent with our previous study on ifenprodil (Poleszak et al. 2013, 2014), which selectively binds to the NR1/NR2B receptor subtype (Williams 2009). We demonstrated that ifenprodil has an antidepressant effect in the FST at the same dose range (20–40 mg/kg) (Poleszak et al. 2013). Shortening the duration of immobility observed in both studies using traxoprodil and ifenprodil was not associated with the increase of spontaneous locomotor activity (Poleszak et al. 2013). Moreover, the highest dose used by us exerted an effect similar to the action of imipramine administered at an active dose (30 mg/kg). Based on the dose-effect examination, the dose of traxoprodil for further testing was selected.

Recent studies indicate that ifenprodil co-administered with antidepressant agents with distinct pharmacological profiles, each given at ineffective doses, produced a significant antidepressant-like effect in the FST (Ghasemi et al. 2009; Poleszak et al. 2014). A similar effect on the duration of the immobility time was observed in animal studies in which low doses of other NMDA receptor antagonists were administered concomitantly with antidepressants in the following groups: TCA – imipramine, SSRI – fluoxetine, SNRI – reboxetine, and a selective serotonin reuptake enhancer (SSRE) – tianeptine (Maj et al. 1992a, b; Poleszak et al. 2011, 2013, 2014) (Pruus et al. 2010; Rogóż et al. 2002, 2004).

After the first experiment with imipramine (15 mg/kg), the obtained results suggested that traxoprodil may intensify activity of antidepressant drugs whose mechanism of action is related to the effects of both serotonergic and noradrenergic transduction. Therefore, in the subsequent stages of research, the effect of traxoprodil on the action of antidepressant drugs which affect selectively particular neurotransmitter systems has been determined. From SSRIs, fluoxetine (5 mg/kg) and escitalopram (2 mg/kg) have been selected for examination. Reboxetine (2.5 mg/kg) has been chosen as a representative of the SNRIs group. Traxoprodil did not affect the antidepressant activity of reboxetine, but potentiated the effect of all used SSRIs. The target point of action of reboxetine is pre- and postsynaptic adrenergic receptors (Hajos et al. 2004). Its mechanism of action is associated with the selective inhibition of the norepinephrine transporters (NET), which leads to an increased availability of noradrenalin (NA) around synaptic slots (Eyding et al. 2010). No effect of traxoprodil on the antidepressant action of reboxetine may stem from its mechanism of action, and may suggest no impact of NR2B subunit of NMDA receptors on the functioning of the noradrenergic system. Shortening of the immobility time in the FST in mice, thus the synergism of the antidepressant action, was not observed in research conducted in the same scheme using a concomitant administration of reboxetine and ifenprodil at ineffective doses (Poleszak et al. 2014). It should be noticed, that in the literature there are reports indicating a lack of clinical efficacy of reboxetine (Eyding et al. 2010), which can also explain the absence of synergism between traxoprodil and reboxetine.

The mechanism of antidepressant action of SSRIs is associated with selective activity at the rise of the serotonergic system in the CNS by inhibiting the reuptake of serotonin (5-HT). This mechanism, because of its highly selective nature, helps exclude the direct influence of other neurotransmitters, such as NA or dopamine (DA). When it comes to fluoxetine, the fact that it is considered as a selective inhibitor of the NMDA receptor subunit GluN2B could be important (Kiss et al. 2012). The effect of traxoprodil on the antidepressant-like action was marked the strongest for escitalopram, which is the most selective compound of the currently available SSRIs (Montgomery et al. 2001). It is worth remembering that behavioral effects observed in the FST were not associated with the increase in spontaneous locomotor activity of animals. The results obtained in these studies are in line with ongoing research of other authors who have shown the direct interaction between the glutamatergic and serotonergic systems. It has been demonstrated that as a result of the NMDA receptor inhibition increasing the level of 5-HT in CNS neurons was observed (Löscher et al. 1993). Furthermore, non-competitive NMDA receptor antagonists (phencyclidine and MK-801) also enhance the serotonin level in the CNS (Martin et al. 1997; Yan et al. 1997). The synergistic interactions between some NMDA antagonists and the antidepressant drugs whose mechanism of action is associated with serotonergic transduction (Maj et al. 1992a, b; Poleszak et al. 2005, 2007b, 2014; Szewczyk et al. 2002, 2009), and no such interaction in the case of drugs that act selectively on noradrenergic transduction (reboxetine) were noticed (e.g., Poleszak et al. 2007b; Pontieri et al. 1995; Szewczyk et al. 2009).

In order to verify the role of the serotonergic system in the antidepressant-like effect of traxoprodil, serotonergic lesion was performed. A 3-day-long administration of p-chloro- phenylalanine (p-CPA), a compound inhibiting the activity of tryptophan hydroxylase (an enzyme that plays a key role in the biosynthesis of 5-HT in CNS neurons) (O’Leary et al. 2007), did not change the activity of animals in the FST. However, a partial reduction of antidepressant action of traxoprodil (20 mg/kg) was demonstrated. This indicates only the partial participation of the serotonergic system in the antidepressant activity of this agent. The obtained results are not entirely consistent with other authors’ outcomes which showed that the blockade of serotonergic system by p-CPA revoke the effect of antidepressant action of other ligands that modulate the NMDA receptor, such as Mg^2+^, Zn^2+^ or d-cycloserine (Poleszak et al. 2007b, 2011; Szewczyk et al. 2009).

The demonstration of a partial role of serotonergic neurotransmission in the action of traxoprodil became the reason to continue studies aimed at clarification of the role of different subtypes of serotonin receptors in its antidepressant effect. Literature data indicate that in the course of affective disorders an increase in sensitivity of 5-HT_2A_ receptors and desensitization of 5-HT_1A_ are observed (Stahl 1994). What is more, some researchers believe that lowering the sensitivity of 5-HT_1A_ receptors is the primary mechanism underlying dysfunction of serotonergic system in major depressive disorder (Cowen 2000). The evidence of this hypothesis may be the fact that during the administration of antidepressants, the sensitivity of 5-HT_1A_ receptors significantly increased (Hensler 2003; Savitz et al. 2009). Therefore, the impact of WAY 100,635 (selective antagonist of the 5-HT_1A_) and ritanserin (selective antagonist of the 5-HT_2A/2C_) (Akhondzadeh et al. 2008; Nappi et al. 1990) on the antidepressant activity of traxoprodil in the FST in mice was examined. The results showed that WAY 100,635 and ritanserin applied at a dose of 0.1 and 4 mg/kg, respectively, did not affect the antidepressant activity of traxoprodil (20 mg/kg). It should be noticed that, in the case of ifenprodil, the significant effect of blockade of the 5-HT_1A_ on its antidepressant activity was demonstrated (Poleszak et al. 2014). In the case of traxoprodil (derivative of ifenprodil) a similar effect was expected. It should be stressed that the influence of above-mentioned substances on the activity of the NMDA receptor ligands (e.g., MTEP, zinc, chromium) was previously observed (Pałucha-Poniewiera et al. 2014; Piotrowska et al. 2008; Szewczyk et al. 2009).

Due to a high probability of interaction between traxoprodil and antidepressants in the pharmacokinetic phase, the concentrations of the tested drugs in murine brain were determined . Traxoprodil is metabolised by the system of cytochrome P450 and influences the activity of izoenzyme CYP2D6 (Johnson et al. 2003), which is engaged in the metabolism of some antidepressants, e.g., imipramine, desipramine, citalopram, fluoxetine, paroxetine or mianserin (Pużyński 2005). When changes occur as a result of action of antidepressant drugs but changes in the levels of these drugs in blood and/or brain are not observed, it may be suspected that the interaction occurs in the pharmacodynamic phase (DeVane 2005). Pharmacodynamic interactions, as opposed to pharmacokinetic ones, do not alter drug concentrations in blood and sites of drug action (DeVane et al. 2002).

Therefore, the data obtained in our studies suggested that the interaction between traxoprodil and all tested antidepressants have a pharmacokinetic character, insomuch as there were significant changes in traxoprodil or tested agents concentrations in murine brain tissue. The increase in antidepressant-like activity of imipramine observed in the FST, was most likely to be the result of pharmacokinetic interaction. It has been shown that traxoprodil significantly enhanced levels of imipramine (and its metabolite) in brains of mice treated concomitantly with imipramine and traxoprodil. In the case of fluoxetine it has been shown that its co-administration with traxoprodil significantly enhances traxoprodil concentration in brain tissue. This may indicate that traxoprodil has an impact on imipramine metabolism and facilitates imipramine/desipramine penetration to the brain, leading to an augmentation in imipramine/desipramine concentration in brain of experimental animals, while in the case of combined injection of traxoprodil and escitalopram an increase in the mice brain concentrations of both traxoprodil and escitalopram was observed. These results suggest that the interaction traxoprodil-escitalopram could have been pharmacokinetic in nature. Because in the group of mice receiving traxoprodil with reboxetine there was no shortening of the immobility time of animals in the FST, reboxetine concentrations in the brain of animals were not assessed. Obviously, it could not be excluded that the observed shortening of the immobility time in the FST after combined administration of traxoprodil and tested drugs is also a result of the interaction in the pharmacodynamic phase (e.g., changes in the concentration of neurotransmitters or receptor level in the CNS). Therefore, it is necessary to expand research to analyse the nature of the occurring interactions in more detail. Pharmacodynamic interactions between traxoprodil and other antidepressant drugs are still worth studying.

Summarizing the results of our research, it should be noticed that selective antagonist of the NR2B subunit of the NMDA receptor – traxoprodil at a dose 20 and 40 mg/kg, exhibits antidepressant-like effect in the FST in mice. Co-administration of traxoprodil with imipramine, fluoxetine or escitalopram, each in subtherapeutic doses, significantly affects the animals’ behavior in the FST and, what is important, these changes are not due to the severity of locomotor activity of animals. The observed effect of traxoprodil is only partially associated with serotonergic system and is independent of the effect on the 5-HT_1A_ and 5-HT_2_ serotonin receptors. The results of an attempt to assess the nature of the interaction between traxoprodil and the tested drugs show that in the case of joint administration of traxoprodil and fluoxetine, imipramine or escitalopram, there are interactions in the pharmacokinetic phase.

The obtained results suggest that the use of traxoprodil together with antidepressants can allow us to lower the doses of antidepressant agents and contribute to a more effective and safer pharmacotherapy of patients suffering from affective disorders.
